# Diffusion weighted imaging in patients with rectal cancer: Comparison between Gaussian and non-Gaussian models

**DOI:** 10.1371/journal.pone.0184197

**Published:** 2017-09-01

**Authors:** Georgios C. Manikis, Kostas Marias, Doenja M. J. Lambregts, Katerina Nikiforaki, Miriam M. van Heeswijk, Frans C. H. Bakers, Regina G. H. Beets-Tan, Nikolaos Papanikolaou

**Affiliations:** 1 Foundation for Research and Technology - Hellas (FORTH), Institute of Computer Science, Computational Biomedicine Lab, Heraklion, Greece; 2 Department of Radiology, the Netherlands Cancer Institute, Amsterdam, The Netherlands; 3 GROW School for Oncology and Developmental Biology – Maastricht University Medical Centre+, Maastricht, The Netherlands; 4 Department of Radiology, Maastricht University Medical Centre+, Maastricht, The Netherlands; 5 Clinical Computational Imaging Group, Centre for the Unknown, Champalimaud Foundation, Lisbon, Portugal; Shanxi University, CHINA

## Abstract

**Purpose:**

The purpose of this study was to compare the performance of four diffusion models, including mono and bi-exponential both Gaussian and non-Gaussian models, in diffusion weighted imaging of rectal cancer.

**Material and methods:**

Nineteen patients with rectal adenocarcinoma underwent MRI examination of the rectum before chemoradiation therapy including a 7 b-value diffusion sequence (0, 25, 50, 100, 500, 1000 and 2000 s/mm^2^) at a 1.5T scanner. Four different diffusion models including mono- and bi-exponential Gaussian (MG and BG) and non-Gaussian (MNG and BNG) were applied on whole tumor volumes of interest. Two different statistical criteria were recruited to assess their fitting performance, including the adjusted-R^2^ and Root Mean Square Error (RMSE). To decide which model better characterizes rectal cancer, model selection was relied on Akaike Information Criteria (AIC) and F-ratio.

**Results:**

All candidate models achieved a good fitting performance with the two most complex models, the BG and the BNG, exhibiting the best fitting performance. However, both criteria for model selection indicated that the MG model performed better than any other model. In particular, using AIC Weights and F-ratio, the pixel-based analysis demonstrated that tumor areas better described by the simplest MG model in an average area of 53% and 33%, respectively. Non-Gaussian behavior was illustrated in an average area of 37% according to the F-ratio, and 7% using AIC Weights. However, the distributions of the pixels best fitted by each of the four models suggest that MG failed to perform better than any other model in all patients, and the overall tumor area.

**Conclusion:**

No single diffusion model evaluated herein could accurately describe rectal tumours. These findings probably can be explained on the basis of increased tumour heterogeneity, where areas with high vascularity could be fitted better with bi-exponential models, and areas with necrosis would mostly follow mono-exponential behavior.

## Introduction

Diffusion weighted imaging (DWI) has an increasing clinical role in the imaging of patients with rectal cancer, especially in the restaging phase after chemoradiation treatment (CRT) [1]. It has been confirmed that DWI improves the diagnostic accuracy when added to conventional T2 sequences for detecting residual disease after CRT [2,3]. The latter has been mainly accomplished by detecting high signals on the high b-value diffusion images by means of visual assessment [1–4], and occasionally by measuring apparent diffusion coefficient (ADC) of these areas [4].

In the era of minimally invasive surgical treatment or even wait and see policies [5,6], it is of paramount importance to develop and validate non-invasive imaging biomarkers that could provide prognostic information on the therapeutic outcome, before initiating the treatment [7]. To serve the latter requirements, an ongoing shift from qualitative evaluation of diffusion images to more quantitative strategies, including measurement of ADC [4,8–9] and tumor volume [10] on high b-value images, is in progress. The most commonly used diffusion related biomarker is the ADC calculated from a mono-exponential model which assumes that the molecular displacement probability function is Gaussian. It has been shown that in several normal tissues, as well as, in malignant, heterogeneous tissues, there is a deviation between the Gaussian diffusion models and the experimental data, noticeable in high b-values which could be attributed to interactions of water molecules with anatomical structures, like cellular membranes. This means, that in the presence of increased tissue heterogeneity the assumption that water displacements can be described by a Gaussian probability function, is no longer valid. In such cases, non-Gaussian models like kurtosis has been shown to fit the data more accurately in the brain [11], breast [12], prostate [13], liver [14] and pancreas [15].

In the current study, four different models (mono- and bi-exponential fitting to Gaussian and non-Gaussian distributions) were applied on data from patients with rectal cancer, to identify which model provided the best performance in terms of fitting quality.

## Materials and methods

### Patients

This study retrospectively assessed nineteen patients who were diagnosed with histologically proven non-mucinous type rectal adenocarcinoma at Maastricht University Medical Center medical center between April 2014 and July 2015. Twelve patients were male, seven females. Median age was 66 (range 45–84 years). Patients were selected from a patient group of n = 28 patients who all underwent a primary staging MRI examination including a dedicated DWI sequence before treatment. Nine patients were excluded because the DW images could not be assessed due to peristaltic motion effects. The study was approved by the Maastricht UMC Medical Review Ethics Committee while informed consent was waived.

### Image acquisition

Diffusion weighted imaging (DWI) using a single shot Spin-Echo Echo Planar Imaging sequence was acquired on a 1.5 T whole-body magnetic resonance scanner (Ingenia, Philips, Best, the Netherlands). For signal reception a 32-channel flexible anterior phased-array coil and built-in posterior coil were used. Seven b-values (number of signal averages) including: 0 (5), 25 (5), 50 (5), 100 (5), 500 (5), 1000 (10) and 2000 (10) s/mm^2^ were acquired. The most important DWI sequence parameters were TR/TE: 4414/79ms; FOV: 320 x 247 mm^2^; acquisition matrix: 176x109; reconstruction matrix: 256x256; slice thickness 5mm and intersection gap 0.4mm. The diffusion gradients were applied in 3 orthogonal axes (tetrahedral scheme), parallel imaging factor was 1.9 and a spectral selective fat saturation pulse was used. The DWI scan time was 08mins and 24s for the acquisition of 20 axial slices. Before the initiation of the diffusion sequence all patients were injected 20mg of Butylscopolamine (Buscopan, Boehringer Ingelheim Pharma, Ingelheim, Germany) to reduce peristaltic motion.

### Image analysis

**Fig 1:**
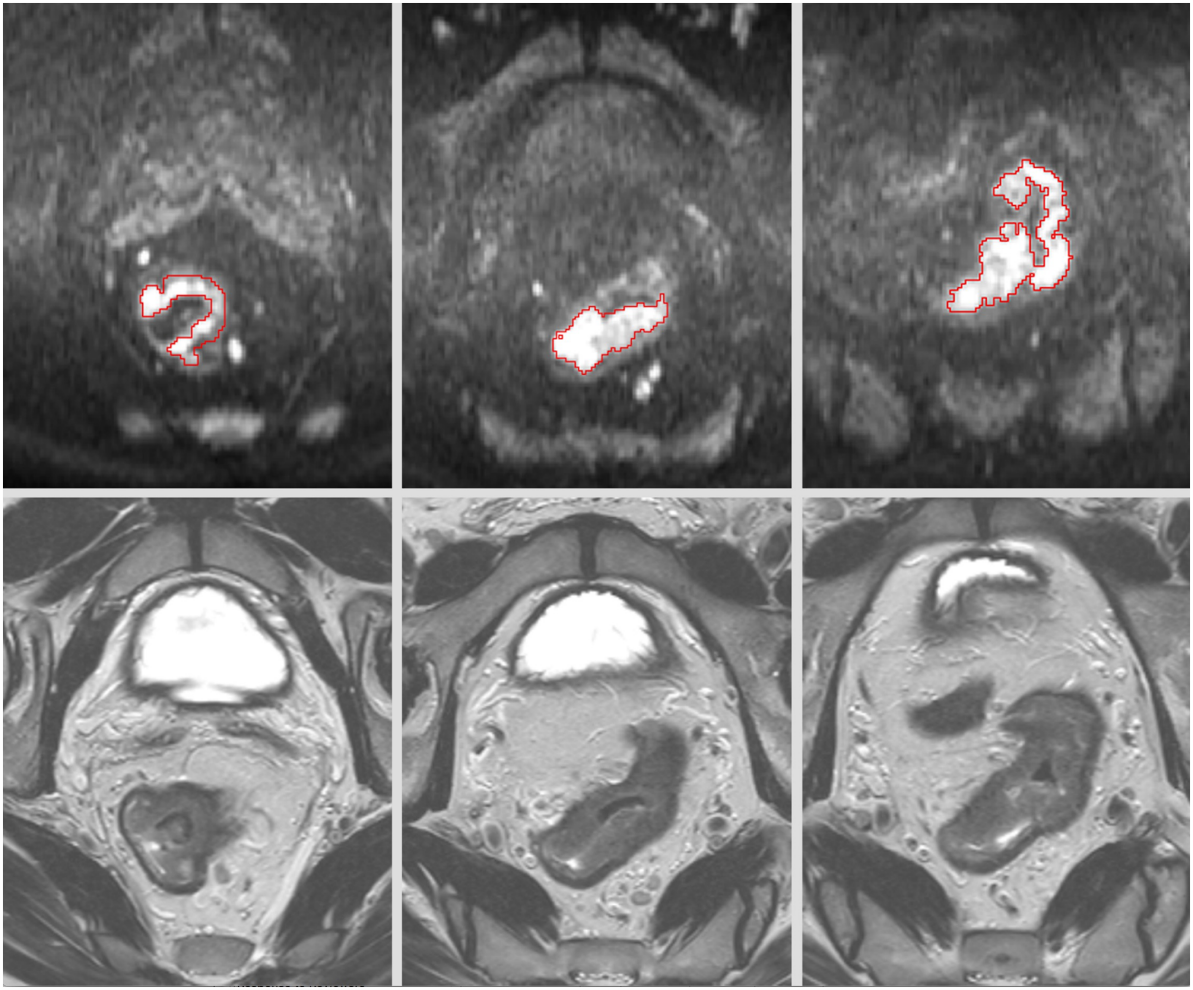
Multiple regions of interest are shown on b1000 diffusion images in a patient with rectal cancer. The tumor was carefully traced based on high signal intensities on b1000 and anatomical information from T2w images.

Diffusion data were post processed with in-house developed software [16] which was able to generate parametric maps of a number of model related parameters. For each patient the tumor was traced manually slice by slice by a trained radiologist with 7 years of experience in rectal MRI. Regions of interest (ROIs) were drawn on the b1000 images, including only the areas with high signal intensity therefore avoiding necrotic parts of the tumors encompassing as much of the tumor volume as possible, avoiding the outmost tumor margins in order to minimize partial volume averaging (Fig 1). The T2-weighted sequences were at the reader’s disposal for anatomical reference.

**Fig 2:**
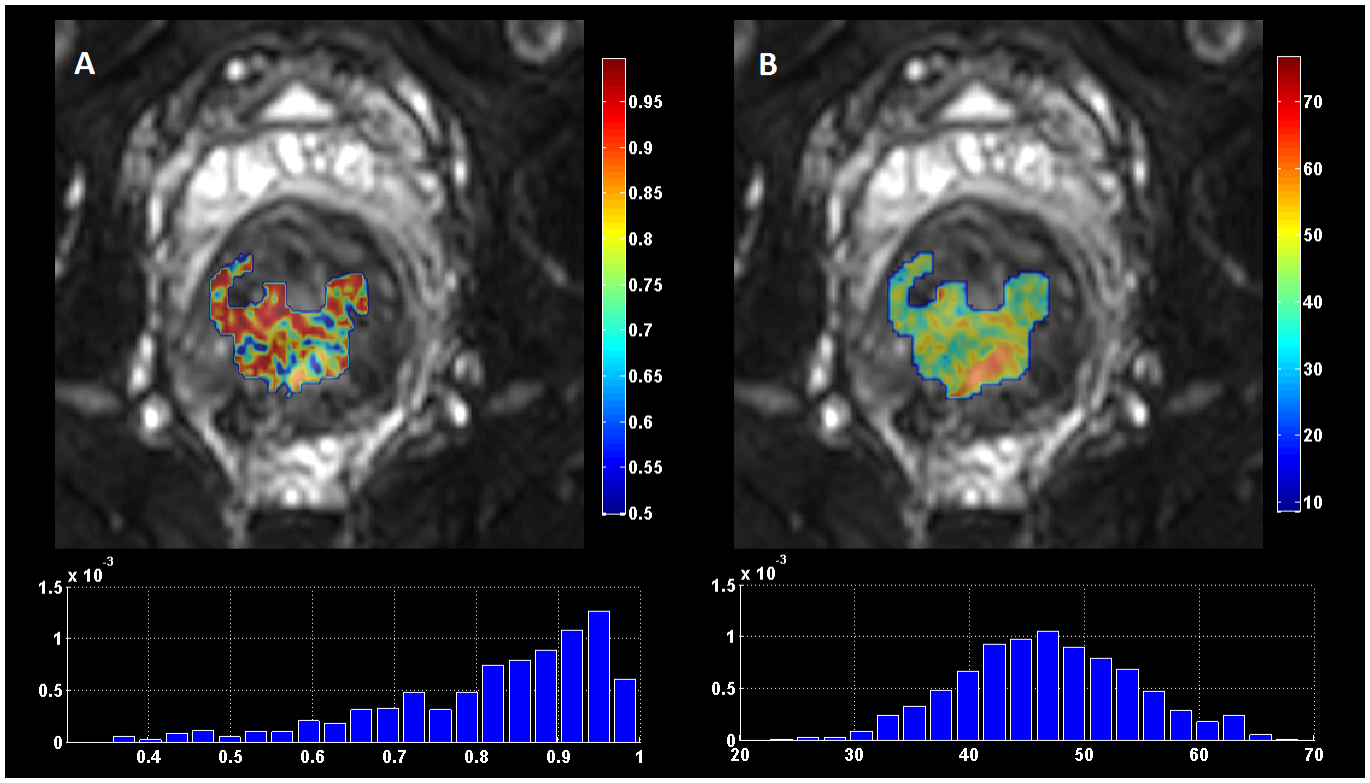
Statistical criteria used to evaluate (A) the quality of fit and (B) model performance. (A) adjusted-R^2^ map and corresponding histogram. (B) Akaike Information Criteria (AIC) map and corresponding histogram.

All pixel values belonging to the tumor were used as input for signal intensity curves as a function of b-value. Model specific curves were graphically overlaid on the data in order to gain insight into each model performance qualitatively. For visual and quantitative evaluation of each approach, statistical criteria permitted direct comparison of the fitting outcome (Fig 2). Signal to Noise Ratio (SNR) maps were calculated on a pixel by pixel basis for each individual b-value based on the following formula that is valid when images obtained with parallel imaging techniques are considered [17]:

$$SNR=\frac{SI_{tumor}}{\sqrt{\frac{2}{4-\pi }sd_{air}}}$$ (1)

where SI_tumor_ was the signal intensity of each tumoral pixel and sd_air_ the standard deviation of a region of interest drown in the air near the anterior abdominal wall.

The analysis was based upon pixels with adequate model compliance to the data. A statistical goodness-of-fit metric relying on the adjusted-R^2^ (adj-R^2^) was applied indifferently of the model, and pixels above a threshold of equal to 50% were included in the analysis. Moreover, in case a ROI showed a very high SNR value at b0 (i.e. 1000), pixels within the ROI with SNR above the 95^th^ quantile of the SNR distribution were also excluded from the analysis as conveying spurious signal. Finally, the analysis was applied individually to each patient, all tumours were visualised on DWI series, and quantitative data were presented as mean ± standard deviation (SD).

### Diffusion signal modelling

**Fig 3:**
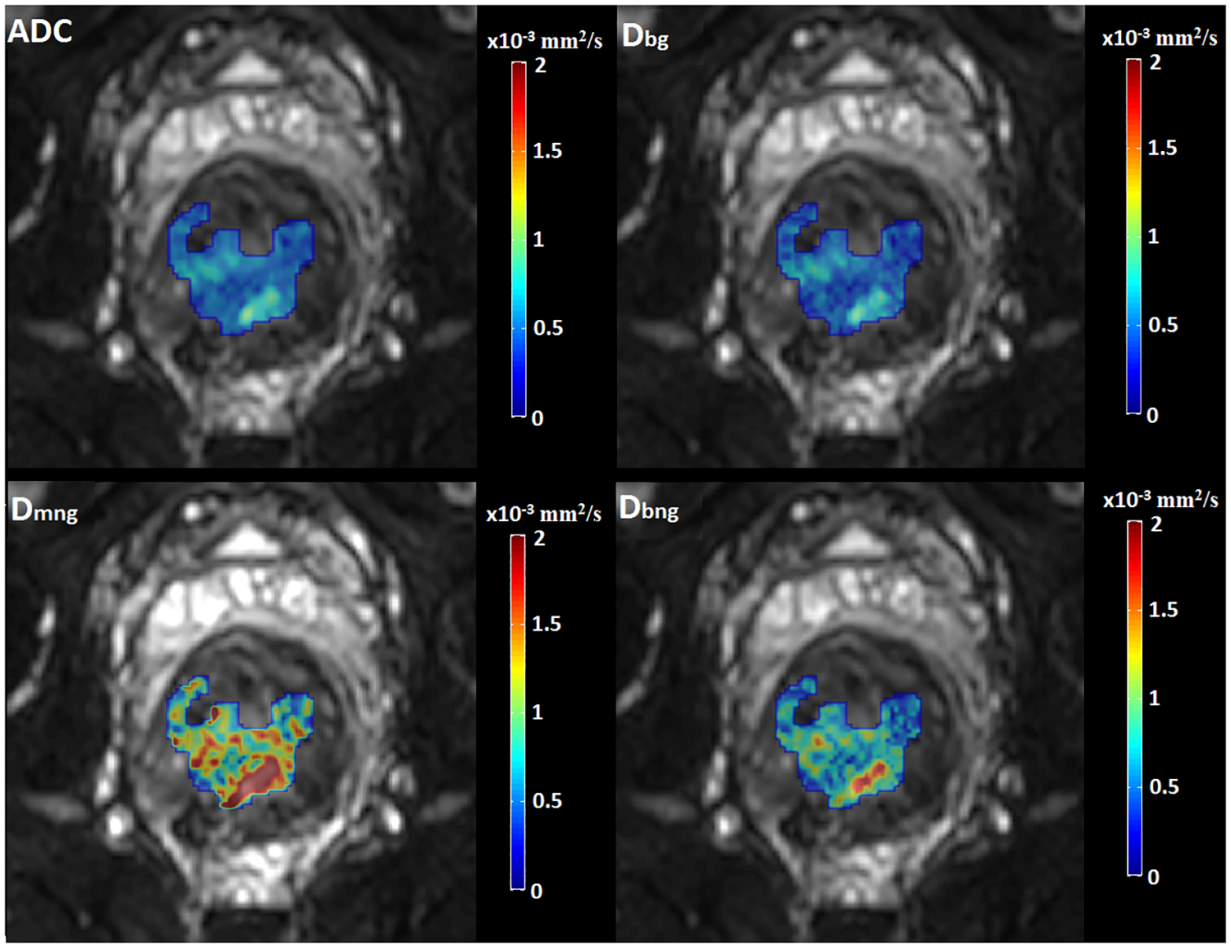
Generated pixel-based ADC and true-diffusion maps from the four examined models. On the upper row ADC and Gaussian true diffusion maps are shown, while on the bottom row non-Gaussian mono and bi-exponential true diffusion maps are visualized. A single slice of the tumor was selected for visualization purposes.

**Fig 4:**
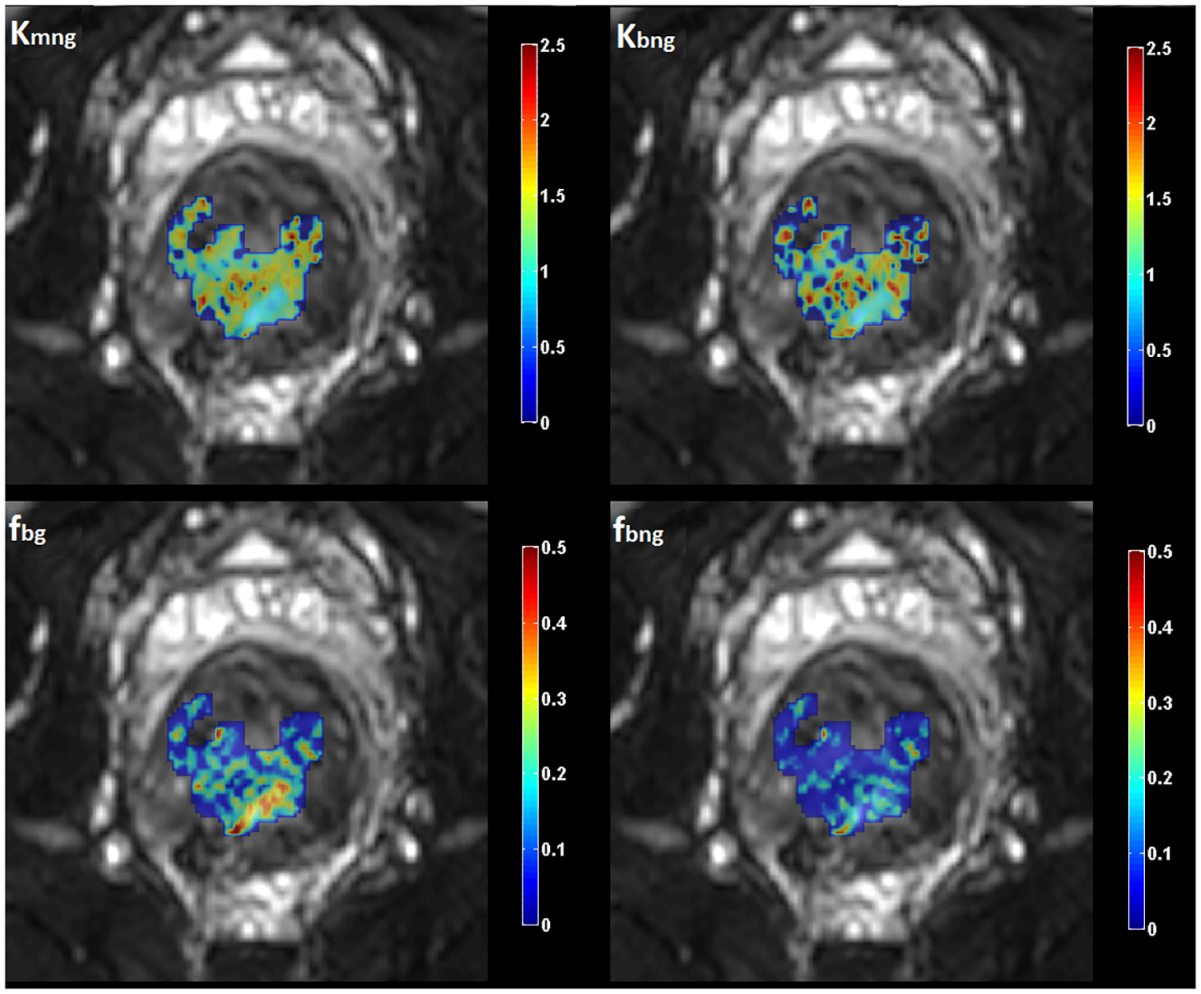
Generated pixel-based kurtosis and micro-perfusion fraction maps from the four examined models. On the upper row non-Gaussian mono and bi-exponential kurtosis maps are shown, while on the bottom row Gaussian and non-Gaussian micro-perfusion fraction maps are visualized. A single slice of the tumor was selected for visualization purposes.

The DWI biomarkers were quantified on a pixel by pixel basis leading to the generation of parametric maps (Figs 3 and 4) of each individual biomarker using the following formulae:

1. ADC from the mono-exponential Gaussian fit, according to:
  $$S\left(b\right)=S_{o}*e^{-b*ADC}$$ (2)
2. D_bg_, D_bg_* and f_bg_ from the bi-exponential Gaussian fit, according to:
  $$S\left(b\right)=S_{o}*\left[\left(1-f_{bg}\right)*e^{-b*D_{bg}}+f_{bg}*e^{-b*D_{bg}^{*}}\right]$$ (3)
3. K_mng_ and D_mng_ from the mono-exponential non-Gaussian fit, according to:
  $$S\left(b\right)=S_{o}*e^{-\left(b*D_{mng}+\frac{1}{6}*b^{2}*D_{mng}^{2}*K_{mng}\right)}$$ (4)
4. D_bng_, f_bng_, D_bng_*, K_bng_ from the bi-exponential non-Gaussian fit, according to:
  $$S\left(b\right)=S_{o}*\left[\left(1-f_{bng}\right)*e^{\left(-b*D_{bng}+\frac{1}{6}*b^{2}*D_{bng}^{2}*K_{bng}\right)}+f_{bng}*e^{-b*D_{bng}^{*}}\right]$$ (5)
  where S(b) is the signal intensity (SI) at a given b-value, S_0_ the SI without any diffusion weighting gradient (b-value equal to 0), ADC the apparent diffusion coefficient, D_bg_ the Gaussian true diffusion coefficient, D_bg_* the Gaussian pseudo-diffusion coefficient, f_bg_ the Gaussian micro-perfusion fraction, D_mng_ is the mono-exponential non-Gaussian diffusion coefficient, K_mng_ the mono-exponential kurtosis coefficient, D_bng_ is the bi-exponential non-Gaussian true diffusion coefficient, D_bng_* is the bi-exponential non-Gaussian pseudo-diffusion coefficient, f_bng_ is the non-Gaussian micro-perfusion fraction and K_bng_ the bi-exponential kurtosis coefficient. The kurtosis coefficient expresses the degree of deviation from the Gaussian distribution and is a dimensionless parameter, whose value may be either 0 (expressing perfect Gaussian distribution) or higher.

All models were applied on multi-slice regions of interest including rectal tumors and their associated biomarkers were derived for every pixel in the region using non-linear fitting techniques. The mean post processing time to generate all diffusion parametric maps was 04:32mins (min: 01:45mins, max: 08:31mins) depending on the number of pixels belonging to the tumor.

Nonlinear Least Squares (NLLS) was applied as a fitting method for calculating the biomarkers from the four different models. NLLS, based on the Levenberg-Marquardt algorithm [18], are minimization problems in mathematics that given initial, lower, and upper bounds for each calculated biomarker (i.e. D_bg_, D_bg_* and f_bg_ for the bi-exponential Gaussian fit) consider the diffusion model by a linear one, and iteratively refine the values of the parameters to reach their optimal values. The following constraints in the initialization values intended to limit possible effect of local minima in the fitting procedure:

1. Mono-exponential Gaussian (MG) model: ADC from 0.1 (10^-3^mm^2^/s) to 4.0 (10^-3^mm^2^/s) with an initial value of 1.5 (10^-3^mm^2^/s).
2. Bi-exponential Gaussian (BG) model: D_bg_ from .1 (10^−3^ mm^2^/s) to 4.0 (10^−3^ mm^2^/s) with an initial value of 1.5 (10^−3^ mm^2^/s), D_bg_* from 10 (10^−3^ mm^2^/s) to 300 (10^−3^ mm^2^/s) with an initial value of 100 (10^−3^ mm^2^/s), and f_bg_ from .05 to .8 with an initial value of .2.
3. Mono-exponential non-Gaussian (MNG) model: D_mng_ from .1 (10^−3^ mm^2^/s) to 4.0 (10^−3^ mm^2^/s) with an initial value of 1.5 (10^−3^ mm^2^/s), and K_mng_ from 0 to 2.5 with an initial value of 1.
4. Bi-exponential non-Gaussian (BNG) model: D_bng_ from .1 (10^−3^ mm^2^/s) to 4.0 (10^−3^ mm^2^/s) with an initial value of 1.5 (10^−3^ mm^2^/s), D_bng_* from 10 (10^−3^ mm^2^/s) to 300 (10^−3^ mm^2^/s) with an initial value of 100 (10^−3^ mm^2^/s), f_bng_ from .05 to .8 with an initial value of .2, and K_bng_ from 0 to 2.5 with an initial value of 1.

### Statistical metrics

Statistical metrics including the R-square (R^2^) and the Root Mean Square Error (RMSE) are frequently used criteria to determine the goodness-of-fit of a model. Studies showed that R^2^ and conclusively metrics that mainly rely on the measurement of the absolute distance between the fitted curve and the given signal have been adequate metrics in nonlinear fitting problems [19–20]. Therefore, the bias-corrected adjusted-R^2^ (adj-R^2^) that accounts for the number of degree of freedom (DOF) was used instead of the R^2^. Both adj-R^2^ and RMSE were included in the analysis to only assess how close the fitted curve was to the measured signal intensity curve, thus providing a strong statistical indicator about the fitting accuracy of the four examined models and the derived diffusion parameters.

Akaike Information Criteria (AIC) [21] and the F-test statistics (F-ratio) [22] were recruited for statistical evaluation of the performance of the four investigated models in terms of model selection. A low value for AIC signifies a good model. However, a direct comparison of the AIC values is meaningless when comparing a series of models [23]. Instead, model selection was performed using AIC Weights. The second metric for model selection relied on a hypothesis test using F-ratio with a 5% level of significance. F-ratio was calculated based on the following equation:

$$F=\;\frac{\left(SSE_{1}-SSE_{2}\right)/SSE_{2}}{\left(DF_{1}-DF_{2}\right)/DF_{2}}$$ (6)

where DF is the degree of freedom given by the number of the b-values minus the number of model parameters, and subscripts 1 and 2 present the simpler and the more complex examined models respectively. F-ratio indicates a pairwise comparison between two candidate models for best fitting, choosing the more complex model (i.e. with subscript 2) in case its p-value is less than the one from the F-table with a 5% level of significance.

Multiple pairwise comparisons were conducted between the four candidate models until the best model was determined. To further extend the statistical analysis, all derived parameters were tested and exhibited non-normal distribution with a p-value of 5% as significant. Therefore, Wilcoxon-Mann-Whitney test was used to disclose any significant differences between all four models.

For adj-R^2^ and AIC Weights, success is measured on the basis of the maximum value of the criterion while the opposite is true for the RMSE where the lowest score pinpoints the most successful model. F-ratio relies on a hypothesis test and no measurements can be displayed.

## Results

### Data fitting and derived parameters

Table 1 summarizes the percentage of fitted pixels within the ROIs of all patients after the thresholding process was applied to all four models. All candidate models effectively fit a wide area of each ROI above the defined threshold, specifically more than 85% of the pixels in all cases. The Gaussian bi-exponential model (BG) fitted the highest number of pixels of each patient with an adj-R^2^ value superior to 95% (for 15 out of 19 patients). SNR of the tumour area for every b-value, expressed as mean ± SD, is also shown on Table 1. An average SNR level above 240 was achieved in the low b-value range (b-value from 0 to 100 s/mm^2^), decaying smoothly to 85 as the b-value increases.

**Table 1:** Pixel percentage of all patients fitted with adj-R^2^ more than 50% and SNR levels at each b-value. Values in parentheses show the number of patients with highest number of pixels fitted by the model.

**Model**	**Pixel Percentages**
MG	93.49 (4/19)
BG	97.93 (15/19)
MNG	94.77 (0/19)
BNG	96.07 (0/19)
**B-value**	**SNR**
0	267 ± 94
25	269 ± 97
50	267 ± 93
100	243 ± 87
500	179 ± 62
1000	129 ± 44
2000	85 ± 28

Abbreviations: adj-R^2^, adjusted-R^2^; SNR, Signal to Noise Ratio; MG, mono-exponential Gaussian; BG, bi-exponential Gaussian; MNG, mono-exponential non-Gaussian; BNG, bi-exponential non-Gaussian.

Table 2 gives an overview of the goodness-of-fit of the four candidate models, where the adj-R^2^ and especially the RMSE indicate that the two most complex models, the BG and the BNG, exhibit the best fitting performance. In case of the adj-R^2^, the BG model showed the best fitting performance in fourteen out of nineteen patients whereas using RMSE the most complex model (BNG) best fitted tumor areas from all patients.

**Table 2:** Derived goodness-of-fit parameters from the four models, expressed as mean ± SD.

Model	adj-R^2^ (%)	RMSE
MG	83.0 ± 19.1 (0/19)	35.0 ± 22.6 (0/19)
BG	92.7 ± 11.6 (14/19)	16.8 ± 12.9 (0/19)
MNG	86.7 ± 17.9 (2/19)	26.1 ± 20.4 (0/19)
BNG	91.3 ± 16.7 (3/19)	13.3 ± 13.3 (19/19)

Abbreviations: adj-R^2^, adjusted-R^2^; RMSE, Root Mean Square Error; MG, mono-exponential Gaussian; BG, bi-exponential Gaussian; MNG, mono-exponential non-Gaussian; BNG, bi-exponential non-Gaussian.

Mean ± SD of each derived parameter from the four examined models are presented in Table 3 for all tumoral pixels. In a significant number of patients (12 out of 19) the ADC from the MG and the D_bng_ from the BNG model showed a statistical dependence according to the Wilcoxon-Mann-Whitney test (p-value higher than 5%). On the contrary, no statistical significant differences (p-value less than 5%) were found between micro-perfusion, micro-perfusion fraction and kurtosis related parameters, respectively.

**Table 3:** Derived DWI parameters from the four models, expressed as mean ± SD.

Model	Parameters	Mean ± SD
MG	ADC	1.006 ± 0.605 (x10^-3^ mm^2^/s)
BG	D_bg_	0.612 ± 0.218 (x10^-3^ mm^2^/s)
	D_bg_*	46.76 ± 84.79 (x10^-3^ mm^2^/s)
	f_bg_	0.186 ± 0.128
MNG	D_mng_	1.459 ± 0.758 (x10^-3^ mm^2^/s)
	K_mng_	1.005 ± 0.465
BNG	D_bng_	0.882 ± 0.391 (x10^-3^ mm^2^/s)
	D_bng_*	87.27 ± 114.2 (x10^-3^ mm^2^/s)
	f_bng_	0.127 ± 0.110
	K_bng_	0.839 ± 0.598

Abbreviations: MG, mono-exponential Gaussian; ADC, apparent diffusion coefficient (10^−3^ mm^2^/s); BG, bi-exponential Gaussian; D_bg_, Gaussian true diffusion (10^−3^ mm^2^/s); D_bg_*, Gaussian pseudo-diffusion (10^−3^ mm^2^/s); f_bg_, Gaussian micro-perfusion fraction; MNG, mono-exponential non-Gaussian; D_mng_, mono-exponential non-Gaussian diffusion (10^−3^ mm^2^/s); K_mng_, mono-exponential kurtosis; BNG, bi-exponential non-Gaussian; D_bng_, bi-exponential non-Gaussian true diffusion (10^−3^ mm^2^/s); D_bng_*, bi-exponential non-Gaussian pseudo-diffusion (10^−3^ mm^2^/s); f_bng_, non-Gaussian micro-perfusion fraction; K_bng_, bi-exponential kurtosis coefficient.

### Model selection

The statistical analysis was performed with respect to the model selection criteria of the AIC Weights and the F-ratio. To obtain a more detailed insight into the performance of the four examined models, the number of pixels that were attributed to a certain criterion according to the best fitting were calculated and depicted in Table 4. The majority of pixels from each ROI were in general assigned to the model proven to be the most successful. In case of the Akaike Weights and the F-ratio, most of the pixels seemed to be better characterized by the mono-exponential Gaussian decaying curve and the ADC parameter. In the same twelve out of nineteen patients, most of the pixels from the tumor area were better characterized by the MG model either using AIC Weights or F-ratio. However, the F-ratio led to a more balanced distribution of the overall number of pixels from all ROIs to the four examined models when compared to the AIC Weights. The BG model was the second most successful in both criteria, and non-Gaussian behavior was observed in almost 37% and 7% of pixels when F-ratio and AIC Weights were calculated, respectively.

**Table 4:** Percentage of pixels best fitted from a specific model according to the AIC weights and F-ratio.

Model	AIC Weights	F-ratio
MG	52.50 (12/19)	33.29 (12/19)
BG	40.53 (7/19)	29.71 (2/19)
MNG	3.11 (0/19)	24.17 (4/19)
BNG	3.86 (0/19)	12.83 (1/19)

Abbreviations: AIC, Akaike Information Criteria; F-ratio, F-test statistics; MG, mono-exponential Gaussian; BG, bi-exponential Gaussian; MNG, mono-exponential non-Gaussian; BNG, bi-exponential non-Gaussian.

The statistical analysis was extended in terms of the SNR calculations to assess any potential influence they had on the model selection process. The mean value of SNR for every pixel of all ROIs was calculated for the subgroup of low (0, 25, 50, 100 s/mm^2^) and high (500, 1000, 2000 s/mm^2^) b-values, respectively. The corresponding min-to-max SNR ranges for each group were in turn divided into 8 sub-regions of equal width in which all models were individually tested with respect to the aforementioned analysis criteria. The scope of this analysis was focused in testing the level of stability each model performance shows at different levels of SNR. Table 5 shows the percentage of pixels best fitted by each model at each SNR interval for the low and the high b-value range according to the F-ratio, respectively. A consistent behavior, in terms of which model best fitted the most pixels, was shown in the low, high and overall b-value range (Table 4) with minor alterations to the percentage differences along the SNR intervals. Similar results in terms of stability were obtained in case of the AIC Weights.

**Table 5:** Percentage of pixels best fitted from a specific model according to the F-ratio at each SNR range. SNR distinct intervals were calculated from the low and the high b-values.

B-value range	SNR intervals	MG	BG	MNG	BNG
LOW	[51.3 170)	39.64%	31.53%	20.82%	8.00%
	[170 202)	33.99%	32.15%	22.61%	11.25%
	[202 228)	31.23%	31.72%	22.96%	14.09%
	[228 248)	28.39%	30.80%	26.69%	14.12%
	[248 271)	29.04%	28.99%	26.77%	15.20%
	[271 301)	30.99%	30.50%	25.09%	13.41%
	[301 356)	33.42%	28.61%	23.96%	14.01%
	[356 733]	39.62%	23.39%	24.45%	12.55%
HIGH	[31.1 89.2)	30.21%	30.45%	25.01%	14.33%
	[89.2 102.6)	30.61%	28.77%	26.61%	14.01%
	[102.6 114.9)	30.21%	28.07%	27.45%	14.28%
	[114.9 125.8)	29.96%	28.58%	28.23%	13.22%
	[125.8 136.4)	30.53%	29.18%	26.88%	13.41%
	[136.4 148.8)	32.23%	30.48%	24.31%	12.98%
	[148.8 171.5)	37.99%	32.94%	18.85%	10.22%
	[171.5 517.7]	44.59%	29.23%	16.01%	10.17%

Abbreviations: F-ratio, F-test statistics; SNR, Signal to Noise Ratio; MG, mono-exponential Gaussian; BG, bi-exponential Gaussian; MNG, mono-exponential non-Gaussian; BNG, bi-exponential non-Gaussian.

**Fig 5:**
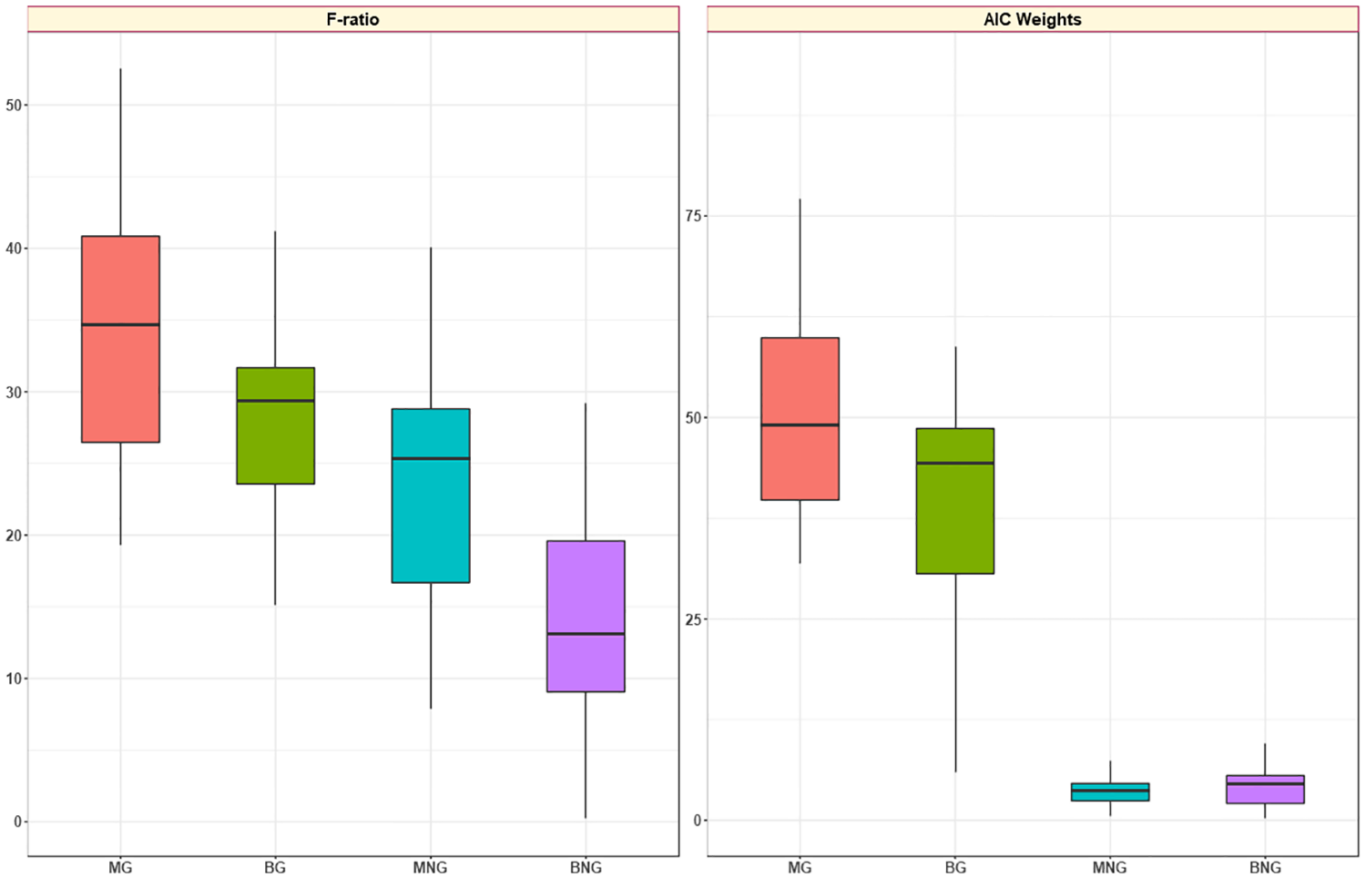
Boxplots showing patient-based percentage of pixels best fitted from a specific model. Percentage of pixels best fitted from a specific model according to the two chosen criteria for model selection.

In order to conclude to a single model that achieves best fitting performance for each patient individually, an average percentage of pixels best described by each model was first calculated for each patient and then summarized as shown in Table 6 and Fig 5. The purpose of evaluating fitting performance for each patient individually was to remove dependence from lesion size (number of pixels assigned as tumor) and more importantly to decide upon the most appropriate model for personalized data fitting. The results shown equivalent results to those presented in Table 4.

**Table 6:** Patient-based percentages of pixels best fitted from a specific model according to the AIC weights and F-ratio.

Model	Metric	# of Patients	Pixel Percentages
MG	F-ratio	12/19	34.4 ± 10.4
	AIC	12/19	52.5 ± 16.9
BG	F-ratio	2/19	28.7 ± 9.2
	AIC	7/19	39.2 ± 14.6
MNG	F-ratio	4/19	23.3 ± 9.1
	AIC	0/19	4.0 ± 2.5
BNG	F-ratio	1/19	13.6 ± 7.3
	AIC	0/19	4.3 ± 2.5

## Discussion

Diffusion imaging is gaining increasing attention for rectal cancer imaging not only qualitatively but also quantitatively [8,24]. The predictive value of ADC in assessing the treatment outcome has already been demonstrated by a limited number of studies [25–27]. In the vast majority of these studies a mono-exponential Gaussian algorithm is used in order to extract quantitative information. In the presented study, we acquired multiple b-values located on the low and very high range, in order to bring out micro-perfusion contamination and deviations from the Gaussian behavior [28].

A comprehensive statistical analysis was conducted to assess fitting quality of each model using the adj-R^2^ and RMSE. As reported in the literature, metrics that rely on the measurement of the absolute distance between the fitted curve and the acquired diffusion signal tend to favour the most complex models [19–20]. Statistically, a complex model like BNG would better fit the data than a simple model like MG causing overfitting and consequently false model selection in some cases. Therefore, model selection analysis was based on the AIC Weights and F-ratio showing contradictory to the goodness-of-fit metrics results.

**Fig 6:**
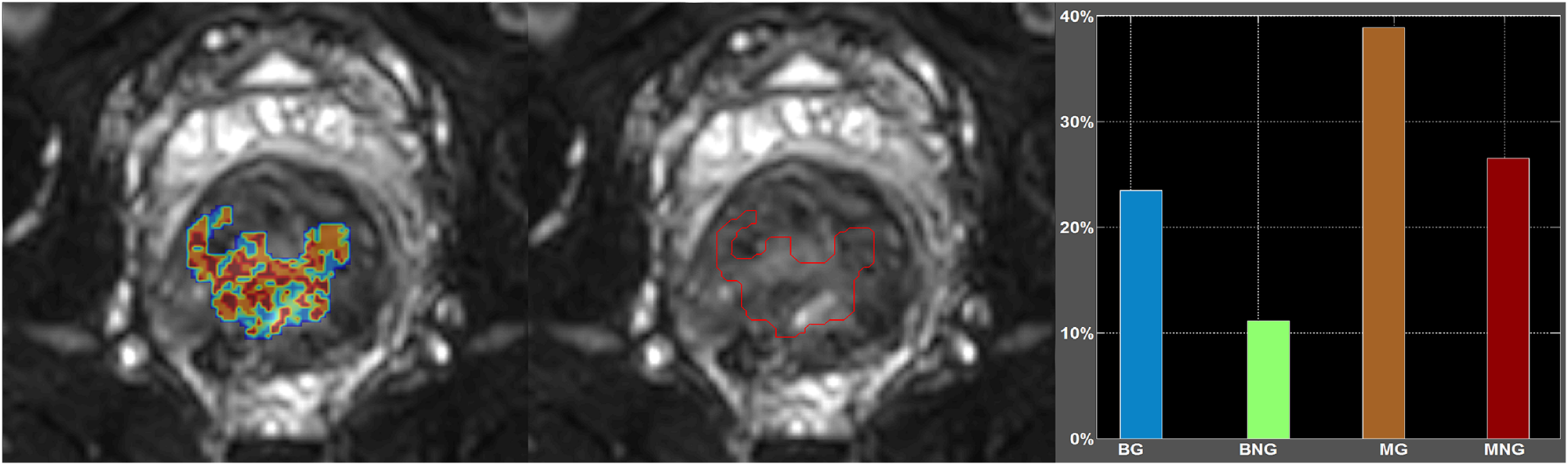
Composite diffusion model parametric map on a patient with rectal cancer. All four models were applied on a pixel by pixel basis. Mono and bi-exponential Gaussian and non-Gaussian models were presented with different colors using F-ratio for model selection.

A trend was distinguished when considering both chosen criteria (i.e. AIC Weights and the F-ratio) for model selection, which evince the MG as the most reliable fitting algorithm. However, a high heterogeneity of all ROIs was prominent and statistically presented through the percentage of pixels best fitted by each of the four models. Table 4 shows that the AIC Weights and especially the F-ratio showed a balanced and smoothed distribution of the pixels better fitted by the models. Extending the current analysis into eight different SNR intervals, Table 5 depicted that model performance was not influenced by the SNR of the tumor area. These results were further confirmed when average percentages of all pixels of the tumor area of each patient that best fitted by each model were summarized in Table 6 and Fig 5. These preliminary findings suggest that in heterogeneous tissue areas, a single model cannot quantitatively describe all the underling anatomical and functional diversity. Therefore, a composite diffusion model (CDM) map could be useful to reflect tumour heterogeneity by presenting the most accurate diffusion model on a pixel by pixel basis (Fig 6).

**Fig 7:**
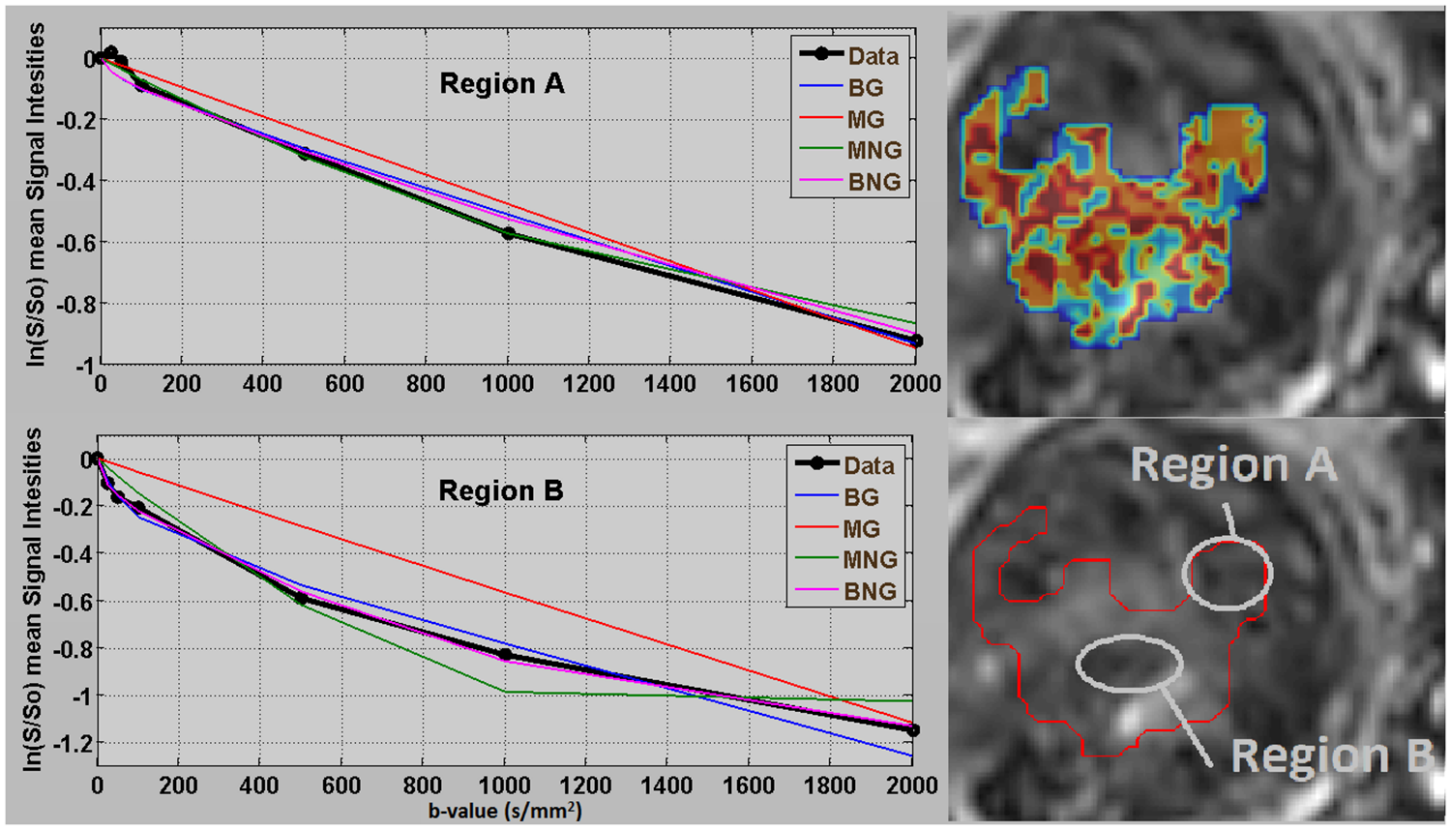
Measured mean S/S0 and fitted curves in semi-log from two different areas within a ROI of a patient with rectal cancer. All four models, represented with different colors, were applied on a pixel by pixel basis to two different areas showing mono- (Region A) and bi-exponential (Region B) behavior respectively.

Measured mean signal and fitted curves from the four examined models, applied to two different areas (Region A and B) from Fig 6, were depicted in the following figure (Fig 7). As seen from Fig 7, “Region A” which was classified according to the model selection criteria as an area with a mono-exponential behavior was better fitted by the MG model compared to “Region B” which was better described by BG and BNG.

In the present study, when measuring the signal decay in tumoral pixels, we observed a significant deviation not only in the low b-value area that can be explained in the basis of micro-perfusion contamination, but in the high b-value area due to probably increased tumoral heterogeneity. Although the signal to noise ratio (SNR) decreases considerably with higher b-values, our acquisition protocol with asymmetric averaging scheme and utilization of state of the art reception RF coils resulted in high SNR values of the tumor even in the images obtained with a b value of 2000 s/mm^2^.

We must acknowledge several limitations in the current study. The relative small number of patients is a potential limitation although for the purpose of the study it was considered adequate. Another limitation was the absence of the application of motion correction techniques between the different b-values. Motion correction in the rectum is not a trivial problem often requiring the development of sophisticated elastic deformation algorithms, which was not under the scope of this study. However, proper administration of antiperistaltic drugs just before the diffusion acquisition minimized such motion-related issues.

In conclusion, the current study indicates that there is no single diffusion model that can describe rectal tumors accurately. Our results suggest that a combination of different models can add value for describing tumor heterogeneity quantitatively in the context of composite diffusion model maps. These findings probably can be explained on the basis of increased tumoral heterogeneity in these lesions, where areas with high vascularity could be better fitted by bi-exponential models, and areas with necrosis would mostly follow mono-exponential behavior.

## Supporting information

S1 Dataset The dataset used in this analysis is available in the file S1_Dataset.zip. The derived DWI parameters from the four examined models and the statistical analysis results are provided in csv format.
(ZIP)
