# Correction: Diffusion weighted imaging in patients with rectal cancer: Comparison between Gaussian and non-Gaussian models

**DOI:** 10.1371/journal.pone.0190290

**Published:** 2017-12-20

**Authors:** Georgios C. Manikis, Kostas Marias, Doenja M. J. Lambregts, Katerina Nikiforaki, Miriam M. van Heeswijk, Frans C. H. Bakers, Regina G. H. Beets-Tan, Nikolaos Papanikolaou

The affiliation for the sixth author is incorrect. Frans C. H. Bakers is not affiliated with #3 but with #4 Department of Radiology, Maastricht University Medical Centre+, Maastricht, The Netherlands.
