# Correction: Diffusion weighted imaging in patients with rectal cancer: Comparison between Gaussian and non-Gaussian models

**DOI:** 10.1371/journal.pone.0196262

**Published:** 2018-04-17

**Authors:** Georgios C. Manikis, Kostas Marias, Doenja M. J. Lambregts, Katerina Nikiforaki, Miriam M. van Heeswijk, Frans C. H. Bakers, Regina G. H. Beets-Tan, Nikolaos Papanikolaou

One affiliation for the second author is not indicated. Kostas Marias is also affiliated with: Department of Informatics Engineering, Technological Educational Institute of Crete, Heraklion, Greece.
